# Impact of the Drying Procedure and Botanical Origin on the Physico-Chemical and Potentially Bioactive Properties of Honey Powders

**DOI:** 10.3390/foods12213990

**Published:** 2023-10-31

**Authors:** Leire Cantero, Lara González-Ceballos, Saúl Vallejos, Guillermo Puertas, Miguel A. Fernández-Muiño, M. Teresa Sancho, Sandra M. Osés

**Affiliations:** 1 Department of Biotechnology and Food Science, University of Burgos, Plaza Misael Bañuelos s/n, 09001 Burgos, Spain; lcantero@ubu.es (L.C.); lgceballos@ubu.es (L.G.-C.); gpl1001@alu.ubu.es (G.P.); smoses@ubu.es (S.M.O.); 2 Department of Chemisty, University of Burgos, Plaza Misael Bañuelos s/n, 09001 Burgos, Spain; svallejos@ubu.es; 3 Faculty of Sciences, University of Burgos, Plaza Misael Bañuelos s/n, 09001 Burgos, Spain

**Keywords:** honey powder, freeze drying, vacuum drying, potentially bioactive properties

## Abstract

This study was aimed at researching the impact of the drying procedure (using the most appropriate honey–maltodextrin concentration for each drying technique) and botanical origin of honey on the physicochemical and potentially bioactive properties of honey powders that were made using maltodextrin as a carrier. The research was carried out with thyme, lavender, vetch and multifloral honey dehydrated using vacuum drying and freeze drying. The analysed parameters were moisture, water activity, colour, glass transition temperature, powder recovery, hygroscopic index and rate, tapped density, solubility, and phenolics as well as antiradical (ABTS^•+^, ROO^•^, ^•^OH and O_2_^•−^), anti-inflammatory and antimicrobial (against *Staphylococcus aureus*, *Escherichia coli* and *Listeria monocytogenes*) activities. Freeze drying provided the highest recoveries. Powders obtained using freeze drying showed higher moisture and solubility as well as lower glass transition temperature, density and hygroscopicity than those obtained using vacuum drying. Hygroscopicity, glass transition temperature and antimicrobial activity against *St. aureus* depended on the drying procedure–honey concentration. Colour, anti-O_2_^•−^ activity and antimicrobial activity against *L. monocytogenes* depended on the botanical origin of the raw honey. Moisture, solubility, density, total phenolic content, anti-ABTS^•+^ and anti-ROO^•^ activities as well as anti-inflammatory activity and antimicrobial activity against *E. coli* depended on the drying procedure–honey concentration and botanical origin.

## 1. Introduction

Honey is a natural sweetener with a long history of use in human nutrition. Honeys from different botanical/geographical origins are highly liked by consumers for their unique flavours and nutrient compositions. In Spain, heather, thyme, vetch, lavender and some multifloral honeys are highly valued. Food packers and industries appreciate honey because of its potential health benefits that are generally related to micro components such as polyphenols, organic acids, enzymes, and amino acids, among others, that may provide antioxidant, antimicrobial, anti-inflammatory and immunostimulant actions [1,2,3]. Consumers associate honey with nature and ecology [4]. An important drawback regarding honey’s potential use in food, pharmaceutical and cosmetic industries is the high viscosity and stickiness of this foodstuff, which creates multiple problems mainly related to honey’s tendency to crystallize due to its high carbohydrate content. Therefore, alternatives to raw honey are being researched nowadays, among which honey powder (P) is one of the most important options [5]. Honey powder has been used as an ingredient in several food products, such as bread, cookies or meat marinade [6,7,8]. The addition of up to 15% of honey powder to food products inhibited the development of oxidative compounds in cooked turkey meat [6]. The addition of honey powder also improved dough rheology and textural properties in bread [7]. Kilinç and Demir [8] made cookies replacing sugar with honey powder, increasing the mineral content and total phenolic compounds. López-Patiño [9] used honey powder in grilled meat, slowing down the microbial growth of enterobacteria and increasing the shelf life of the product.

P has many advantages compared to honey, such as ease of packaging, transportation, and storage operations due to volume and weight reduction, and combination with other dry products [1,10]. P is a hygroscopic powder, which is difficult to obtain because of its composition and structure. Honey fructose (38–40%) and glucose (33–35%), together with other components, are responsible for honeys’ low glass transition temperatures (*T*_g_), around −27 and −51 °C [3,11], which make it impossible to obtain P without the addition of carriers [12,13]. Arabic gum, dextrins, maltodextrins and whey protein concentrate are common carriers, acting as emulsifying agents, anti-caking agents and high-molecular-weight filling materials. Other carriers that are currently being successfully used are bee pollen together with Arabic gum [14] and rice and pea protein [15]. They increase the *T*_g_ of the mixtures, maintaining the stability of the powders with high sugar content and avoiding the transition of the material from a vitreous to a rubbery state. Carriers minimize the main problems during drying, such as stickiness and drying difficulties [16,17]. However, the use of large amounts of drying carriers increases the production costs and can alter the original flavour of raw honeys and the resulting powders, with the risk of consumer disapproval [18].

Spray drying (SP) is the most used method for making P due to its short drying contact time, which prevents the degradation of flavours, colours and nutrients. SP provides powders with effective reconstitution characteristics, storage stability and low water activity, but with decreased antioxidant activity and total phenolic content [19]. Among the various drying technologies, there are other less-used procedures, such as vacuum drying (VC), microwave vacuum drying, and freeze drying (FZ). The mentioned techniques are of great interest since they reduce changes due to the high temperature of spray drying [1,2]. VC takes place in the absence of oxygen, which reduces oxidative degradation and has a faster drying rate than SP due to the creation of a foamy or swollen structure in the powder [3,20]. As a result of the mild drying conditions (low temperature in comparison with SP, and reduced pressure), high and valuable amounts of thermosensitive nutrients are preserved [3]. FZ is slower and more expensive than other procedures [3]. However, FZ (carried out in the absence of oxygen and using low drying temperatures) shows some advantages over other drying techniques, such as a high content of volatile compounds, retention of heat-sensitive compounds and flavours, preservation of structure, low damage from non-enzymatic or oxidative reactions, and maintenance of nutritional and bioactive properties [2].

In previous studies, we verified that ling-heather honey powders (LHP) obtained via VC and FZ using only maltodextrin (MD) as a carrier showed higher recoveries (88–98%) and better sensory characteristics than the powders obtained via SP and/or using other carriers [11]. LHP also exhibited interesting antiradical, antimicrobial and anti-inflammatory activities, which could help boost the use of these powders [21]. Considering previous research on honey powders, the purpose of this work was to determine the actual impact of both the drying procedure (using the most appropriate honey–maltodextrin concentration for each drying technique) and the botanical origin of honey on the physicochemical and functional characteristics of P made with honeys from different botanical origins and dehydrated by VC and FZ, analysing physicochemical and bioactive properties, as well as antiradical activities.

## 2. Materials and Methods

### 2.1. Materials

Reagents: Maltodextrin (MD) of dextrose equivalent 20 (Calaf Nuances, Barcelona, Spain). Methanol, sodium carbonate, sodium chloride, sodium hydroxide, potassium hydroxide, potassium persulfate, Fe(NH_4_)_2_(SO4)_2_, EDTA, acetic acid, formic acid, p-dimethylaminobenzaldehyde (DMAB), HCl, H_2_SO_4_, H_2_O_2_, Baird Parker agar (BP), Trypone bile x-glucuronide agar (TBX) and egg yolk tellurite sterile emulsion (VWR International Eurolab, part of Avantor, Llinars del Vallés, Spain). Gallic acid, NaNO_2_, Na_2_SO_4_ and catechin (Panreac, Barcelona, Spain). AlCl_3_ and fluorescein sodium salt (Fluka Chemie GmbH, part of Sigma-Aldrich, Buchs, San Galo, Switzerland). Na_2_HPO_4_, NaH_2_PO_4_ (Scharlab, Sentmenat, Spain). Potassium tetraborate, nitro-blue tetrazolium, sodium benzoate and uric acid (Alfa Aesar, part of Thermo Fisher, Kandel, Rheinland-Pfalz, Germany). Nutrient broth No. 2 (NB), brain heart infusion (BHI), agar technical No.2 and Ringer solution (Oxoid, part of Thermo Fisher, Basingstoke, Hampshire, UK). Microinstant Listeria Agar Base (LAB), Listeria enrichment supplement Ottaviani & Agosti and Listeria Selective Supplement (Scharlau S.L., Senmanat, Spain). Folin–Ciocalteu phenol reagent, quercetin, dinitrophenylhydrazine, 2,2′-azino-bis(3-ethylbenzothiazoline-6-sulphonic acid) (ABTS), 6-hydroxy-2,5,7,8-tetramethylchroman-2-carboxylic acid (Trolox), thiobarbituric acid (TBA), xanthine, xanthine oxidase (10110434001), 2,2′-azobis(2-aminopropane)dihydrochloride (ABAP), N-acetil-D-glucosamine (NAG), hyaluronic acid sodium salt from *Streptococcus equi* (53,747), bovine serum albumin (BSA), hyaluronidase from bovine testes type IV-S (1400 U/mL, H3884) (Sigma-Aldrich, part of Merck, Steinheim, Nordrhein-Westfalen, Germany). Water was deionized using a Milli-Q water purification system (Millipore, part of Merck, Bedford, MA, USA).

Equipment: Heraeus Instruments vacutherm (ThermoFisher Scientific, Madrid, Spain); LyoQuest-55 ECO PLUS (Azbil Telstar Technologies, S.L.U., Barcelona, Spain); AquaLab Series 4 TE (Labferrer, Lleida, Spain); Hunter Lab colorimeter (ColorFlex EZ System^®^, Reston, VA, USA); scanning calorimetry with a Q200 DSC analysed equipped with a cooling Intra-Cooler system (TA Instruments, New Castle, DE, USA) at 20 °C/min); 400Bio UV–visible spectrophotometer (Varian, Mulgrave, Vic., Australia; Varioskan LUX microplate reader (ThermoFisher Scientific); sterile 96 well round bottomed polystyrene microtitre plates (Brand, Wertheim, Germany).

### 2.2. Honey Samples and Drying Procedures

Nine samples of honeys from thyme (TH), lavender (LV), vetch (V), and multifloral (M) (Table 1), collected by beekeepers from Castilla y León (Spain), were processed to obtain P. Botanical origins were determined using melissopalynology and sensory analyses [22,23,24,25,26,27].

### 2.3. Honey Powders

The liquid honey was mixed with MD and water. Eighteen samples of P were obtained from the nine honeys and drying carrier (MD) using two drying procedures: VC and FZ. Honey and MD were first separately dissolved in water. Then, they were mixed together by gently stirring with a magnetic stirrer at room temperature. The drying methods and proportions of honey and carrier were selected according to preliminary experiments [11]. VC: mix preparation of 42% (*w*/*w*) of total solid concentration [(honey + MD)/(honey + MD + H_2_O) × 100] and a ratio of honey:MD of 60:40 (57% of honey in dry basis) was poured into a silicon baking cup with 3–4 mm thickness and dried in a lab-scale vacuum oven (Heraeus Instruments vacutherm) at 60 °C with a pressure at 100 mbar for two days and cooling in a desiccator before grounding with a mortar. FZ: The mixtures were prepared at a honey:MD ratio of 75:25 (73% of honey in dry basis), maintaining a total solid concentration of 45%. The mixture was frozen at −30 °C in a silicon mold (3–4 mm) for 5 h and later at −80 °C for 24 h. Later, the samples were dried by freezing in a LyoQuest-55 ECO PLUS for 3 days at 0.057 mbar pressure and immediately ground. Each drying procedure was performed three times for each honey, and the combination of the three powders (obtained using the same procedure for the same honey) was used for the analysis. VC and FZ samples were stored in glass jars in a desiccator containing silica gel until further analyses. Moisture, a_w_, hygroscopicity, solubility, density and colour were analysed during the first week. Total phenols, total flavonoids, antioxidant, anti-inflammatory and antimicrobial analysis were determined within the first month after making the honey powders.

### 2.4. Quality Parameters and Physical Properties of Honey and P

The moisture, water activity (a_w_) and colour of the honeys were determined using the AOAC (969.38 method) [28], Bogdanov [29], Codex Alimentarius Standard for honey [30], Commisssion Internationale de L’elairage [31], Pascual-Maté et al. [32], and Sancho et al. [33] procedures. The water activity and colour parameters L*, a*, and b* of P were determined following the same procedures described for honey using an AquaLab Series 4 TE and a Hunter Lab colorimeter, respectively. Powder recovery, moisture content (gravimetry, drying at 105 °C/4 h), hygroscopic index and rate (1.00 g placed in a desiccator at 25 °C and a saturated NaCl solution during 1 week), tapped density (recording the volume of 2.00 g P in a graduated cylinder after tapping 25 times), solubility (time required for 2.00 g P to dissolve completely in 25 mL distilled water in a 100 mL glass beaker at room temperature, stirring at 890 rpm), and *T*_g_ (scanning calorimetry with a Q200 DSC analysed equipped with a cooling Intra-Cooler system at 20 °C/min) were determined following the Osés et al. [11] procedures briefly described in Supplementary Figure S1.

### 2.5. Analysis of Potentially Bioactive Properties of Honey and P

Phenolic compounds (total phenolic, total flavones/flavonols and total flavanols), antiradical activities against ABTS^•+^, ^•^OH and O_2_^•−^ and anti-inflammatory activity were measured using a 400Bio UV–visible spectrophotometer for absorbance, while fluorescence (antiradical activity against ROO^•^) was measured with a Varioskan LUX microplate reader. Antimicrobial activity against *Staphylococcus aureus* CECT 435, *Escherichia coli* CECT 99 and *Listeria monocytogenes* CECT 934 (Spanish Type Culture Collection, Valencia University, Spain) were assayed using a microdilution method (5–70 g/100 mL), determining the minimal bactericide concentration (MBC) using a sterile 96-well round-bottomed polystyrene microtitre plates. All the bioactive properties of honey and P were performed following the Osés et al. [21] procedures briefly described in Supplementary Figure S2.

### 2.6. Statistical Analysis

All the analyses were performed in triplicate, except *T*_g_ (just once). The results were expressed as averages and standard deviations. One-way analysis of variance (ANOVA) and multifactor ANOVA followed by Tukey´s honestly significant difference test (*p* < 0.05) were used. Pearson correlations and principal component analysis (PCA) (using a data matrix of 18 × 7 for physicochemical properties and a matrix of 18 × 12 for bioactive properties, automatically scaled) were applied to the results. The statistical software Startgraphics Centurion XVIII (Statgraphics Technologies, Inc., The Plains, VA, USA) was used.

## 3. Results and Discussion

Bioactive properties, except % inhibition (anti-O_2_^•−^ and anti-inflammatory activities) and minimal bactericide concentration (MBC), were expressed on dry basis (d.b.) for a proper comparison of honey and P data. Table 2 shows the results of the physicochemical and bioactive properties of the raw honeys.

### 3.1. Powder Recovery

P showed recoveries between 85.82% and 99.89% (Table 3). FZ-P exhibited the highest recoveries (96.60–99.89%), with a 73% honey concentration, regardless of the botanical origin of the honey. VC-P also exhibited high recoveries (85.82–94.89%) with a 57% honey concentration. These yields were similar to those described by Osés et al. [11] (56% honey, MD-VC: 93.34–96.01%; 72% honey, MD-FZ 89.87–95.57%) and Mutlu and Erbas [1] in powders obtained via VC using different carriers (90.95%), in turn being higher than those P obtained by Mutlu and Erbas [1] with MD, regardless of the extraction process (61.95%). Our P recovery was also higher than the value reported by Nurhadi et al. [16] (50% honey, MD-VC: 73%).

### 3.2. Physicochemical Properties

#### 3.2.1. *T*_g_

*T*_g_ is defined as the temperature above which amorphous food material (metastable state) transitions from a glassy solid to a rubbery state, there being significant changes in physical properties and molecular mobility (the position of molecules relative to one another is random) [34,35]. Table 4 shows *T*_g_ of honeys and P obtained using FZ and VC. Honeys’ *T*_g_ ranged from −25.45 to −37.37 °C. *T*_g_ varied depending on both samples’ botanical origins and moisture contents. Regardless, honeys’ botanical origins *T*_g_ decreased when moisture increased, except for TH honeys. Water *T*_g_ was very low, around −138 °C, which allowed it to act as a plasticizer helping reduce the *T*_g_ of mixtures [16]. Powders were characterized by exhibiting two *T*_g_ (*T*_g_1 and *T*_g_2), in comparison with honey, which only exhibited one *T*_g_. The two *T*_g_ of P corresponded to two different polymers formed in the dehydrated samples, *T*_g_1 corresponding to the P polymer and *T*_g_2 corresponding to the carrier polymer [11]. The *T*_g_ values of powders were higher than the *T*_g_ values of honeys. The *T*_g_1 values of FZ-P (12.58–16.50 °C) were lower than those of VC-P (27.75–35.50 °C), confirming the influence of both honey concentration and drying procedure on the *T*_g_ of the final product. VC samples had a lower concentration of honey (57%) compared to FZ samples (73% honey), confirming that *T*_g_ values decrease when the concentration of honey increases and increase when the concentration of the carrier increases, in this case MD, whose *T*_g_ is 205.5 °C [16,18]. Moisture percentages (Table 3) of VC-P (0.702%–1.63%) were lower than those of FZ-P (1.3%0–1.63%), corroborating the influence of moisture on *T*_g_. Since *T*_g_1 of FZ-P was lower than room temperature (25 °C), FZ-P must be stored at refrigeration temperatures within high-barrier packaging [34,36].

**Table 2:** Physicochemical and bioactive properties (mean ± SD) of raw honeys from various botanical origins used to obtain powdered honey (*n* = 3).

	LV1	LV2	LV3	TH1	TH2	V1	M1	M2	M3
Moisture	13.85 ± 0.00 ^f^	14.67 ± 0.00 ^e^	18.74 ± 0.00 ^a^	16.18 ± 0.22 ^b^	15.63 ± 0.24 ^c^	15.09 ± 0.02 ^d^	16.18 ± 0.23 ^b^	16.31 ± 0.00 ^b^	14.67 ± 0.00 ^e^
a_w_	0.506 ± 0.028 ^c^	0.499 ± 0.002 ^c^	0.625 ± 0.038 ^ab^	0.546 ± 0.007 ^bc^	0.557 ± 0.025 ^bc^	0.523 ± 0.044 ^c^	0.694 ± 0.038 ^a^	0.536 ± 0.003 ^c^	0.542 ± 0.035 ^c^
L*	49.87 ± 0.02 ^a^	4.83 ± 0.02 ^d^	25.90 ± 0.04 ^c^	46.92 ± 2.29 ^a^	29.48 ± 0.02 ^bc^	44.33 ± 0.01 ^a^	33.23 ± 0.04 ^b^	28.43 ± 0.01 ^bc^	12.34 ± 0.02 ^d^
a*	0.553 ± 0.012 ^g^	1.11 ± 0.01 ^f^	−0.437 ± 0.031 ^i^	7.14 ± 0.02 ^c^	11.30 ± 0.01 ^a^	0.477 ± 0.023 ^h^	4.89 ± 0.02 ^d^	7.55 ± 0.02 ^b^	3.33 ± 0.03 ^e^
b*	27.43 ± 0.02 ^e^	6.71 ± 0.04 ^i^	19.33 ± 0.06 ^g^	38.06 ± 0.02 ^a^	34.18 ± 0.07 ^b^	23.98 ± 0.05 ^f^	33.26 ± 0.11 ^c^	31.86 ± 0.15 ^d^	16.22 ± 0.10 ^h^
TPC	61.41 ± 1.41 ^d^	100.65 ± 0.65 ^b^	61.64 ± 2.13 ^d^	62.67 ± 0.35 ^d^	131.50 ± 4.34 ^a^	32.73 ± 1.73 ^e^	60.86 ± 1.78 ^d^	76.97 ± 0.82 ^c^	105.50 ± 0.80 ^b^
TPC-E	4.30 ± 0.09 ^e^	12.08 ± 0.14 ^a^	6.34 ± 0.19 ^c^	11.58 ± 0.30 ^a^	9.03 ± 0.35 ^b^	4.48 ± 0.05 ^e^	6.80 ± 0.13 ^c^	8.67 ± 0.08 ^b^	5.71 ± 0.12 ^d^
TFC_C_	2.07 ± 0.06 ^e^	4.05 ± 0.06 ^a^	2.56 ± 0.10 ^d^	4.12 ± 0.17 ^a^	3.05 ± 0.13 ^c^	2.38 ± 0.06 ^d^	1.97 ± 0.05 ^e^	3.62 ± 0.10 ^b^	3.15 ± 0.02 ^c^
TFC_Q_	0.340 ± 0.018 ^g^	1.055 ± 0.012 ^b^	0.529 ± 0.012 ^f^	1.14 ± 0.03 ^a^	0.715 ± 0.014 ^d^	0.608 ± 0.018 ^e^	0.600 ± 0.007 ^e^	0.900 ± 0.018 ^c^	0.367 ± 0.007 ^g^
TEAC (ABTS^•+^)	17.74 ± 5.13 ^e^	106.31 ± 3.40 ^d^	31.33 ± 8.61 ^e^	116.13 ± 3.77 ^cd^	277.72 ± 21.55 ^a^	32.73 ± 0.97 ^e^	95.18 ± 12.99 ^d^	146.13 ± 5.76 ^c^	234.21 ± 22.00 ^b^
AOA (^•^OH)	0.056 ± 0.007 ^bc^	0.052 ± 0.001 ^bc^	0.054 ± 0.011 ^bc^	0.076 ± 0.008 ^a^	0.056 ± 0.006 ^bc^	0.055 ± 0.000 ^bc^	0.062 ± 0.006 ^ab^	0.041 ± 0.002 ^c^	0.062 ± 0.001 ^ab^
ORAC (ROO^•^)	42.76 ± 2.15 ^cd^	34.45 ± 0.56 ^cd^	67.85 ± 16.15 ^ab^	32.98 ± 8.38 ^cd^	37.84 ± 7.46 ^cd^	24.92 ± 7.81 ^d^	53.91 ± 5.00 ^bc^	76.52 ± 2.71 ^a^	31.91 ± 1.55 ^d^
SRS (O_2_^•−^)	45.33 ± 3.06 ^cd^	58.67 ± 2.08 ^a^	25.67 ± 3.51 ^e^	42.67 ± 2.08 ^d^	51.33 ± 0.58 ^bc^	12.50 ± 0.71 ^f^	47.33 ± 1.53 ^cd^	22.00 ± 1.41 ^e^	56.50 ± 3.54 ^ab^
Anti-inflammatory activity	62.60 ± 0.73 ^b^	10.86 ± 0.75 ^g^	64.16 ± 0.81 ^ab^	68.61 ± 0.39 ^a^	48.21 ± 0.71 ^ef^	44.76 ± 5.14 ^f^	59.79 ± 0.05 ^bc^	53.73 ± 0.77 ^de^	54.48 ± 1.76 ^cd^
MBC *St. aureus*	20.00 ± 0.00 ^d^	30.00 ± 0.00 ^c^	30.00 ± 0.00 ^c^	5.00 ± 0.00 ^e^	60.00 ± 0.00 ^b^	70.00 ± 0.00 ^a^	20.00 ± 0.00 ^d^	70.00 ± 0.00 ^a^	20.00 ± 0.00 ^d^
MBC *E. coli*	20.00 ± 0.00 ^c^	20.00 ± 0.00 ^c^	30.00 ± 0.00 ^b^	20.00 ± 0.00 ^c^	40.00 ± 0.00 ^a^	20.00 ± 0.00 ^c^	20.00 ± 0.00 ^c^	40.00 ± 0.00 ^a^	20.00 ± 0.00 ^c^
MBC *L. monocytogenes*	20.00 ± 0.00 ^b^	50.00 ± 0.00 ^b^	60.00 ± 0.00 ^a^	40.00 ± 0.00 ^c^	50.00 ± 0.00 ^b^	53.33 ± 5.77 ^ab^	53.33 ± 5.77 ^ab^	50.00 ± 0.00 ^b^	40.00 ± 0.00 ^c^

LV: lavender honey; TH: thyme honey; V: vetch honey; M: multifloral honey; moisture (%); TPC: total phenols (mg GA/100 g); TPC-E: total phenols in extract (mg GA/100 g); TFC_C_: total flavanols (mg Ct/100 g); TFC_Q_: total flavones/flavonols (mg Q/100 g); TEAC (μmol T/100 g); ORAC (μmol T/g); AOA (mmol UA/100 g); SRS (% inh); anti-inflammatory activity (% inh); MBC from *St. aureus*, *E. coli* and *L. monocytogenes* (%). ^a–i^: different letters show significant differences (*p* < 0.05) among the different botanical origins.

**Table 3:** Physicochemical properties of powdered honey (mean ± SD) from various botanical origins obtained using vacuum (VC) and freeze (FZ) drying using maltodextrin (MD) (*n* = 3).

		LVP1	LVP2	LVP3	THP1	THP2	VP1	MP1	MP2	MP3
Powder recovery	VC	94.62 ± 0.20 ^Ab^	94.89 ± 0.31 ^Ab^	92.33 ± 0.15 ^Bb^	92.07 ± 3.45 ^Bb^	94.24 ± 4.00 ^ABa^	94.20 ± 3.25 ^ABa^	85.82 ± 9.77 ^Bb^	93.83 ± 0.23 ^Bb^	90.41 ± 3.97 ^Bb^
	FZ	97.36 ± 3.53 ^Aa^	96.60 ± 1.92 ^Aa^	99.64 ± 3.21 ^Aa^	99.89 ± 2.21 ^Aa^	98.36 ± 4.46 ^Aa^	98.57 ± 4.21 ^Aa^	98.47 ± 3.62 ^Aa^	99.50 ± 2.52 ^Aa^	97.31 ± 3.07 ^Aa^
Moisture (%)	VC	0.720 ± 0.029 ^Db^	0.971 ± 0.109 ^CDb^	0.740 ± 0.041 ^Db^	1.01 ± 0.10 ^CDb^	1.23 ± 0.03 ^BCa^	1.63 ± 0.12 ^Aa^	0.702 ± 0.108 ^Db^	1.61 ± 0.15 ^Aa^	1.43 ± 0.21 ^ABa^
	FZ	1.32 ± 0.18 ^Aa^	1.47 ± 0.26 ^Aa^	1.30 ± 0.16 ^Aa^	1.36 ± 0.04 ^Aa^	1.53 ± 0.27 ^Aa^	1.33 ± 0.03 ^Ab^	1.32 ± 0.08 ^Aa^	1.48 ± 0.22 ^Aa^	1.63 ± 0.08 ^Aa^
a_w_	VC	0.214 ± 0.001 ^Ea^	0.207 ± 0.002 ^Fb^	0.206 ± 0.002 ^Fb^	0.300 ± 0.003 ^Aa^	0.292 ± 0.002 ^Ba^	0.258 ± 0.002 ^Da^	0.303 ± 0.002 ^Aa^	0.273 ± 0.002 ^Ca^	0.298 ± 0.001 ^Aa^
	FZ	0.210 ± 0.002 ^DEa^	0.224 ± 0.002 ^Aa^	0.211 ± 0.002 ^CDEa^	0.209 ± 0.003 ^Eb^	0.219 ± 0.001 ^ABb^	0.214 ± 0.003 ^BCDEb^	0.216 ± 0.002 ^BCDb^	0.215 ± 0.002 ^BCDEb^	0.217 ± 0.002 ^ABCb^
L*	VC	89.84 ± 0.01 ^Aa^	83.20 ± 0.01 ^Da^	88.77 ± 0.01 ^Ba^	77.75 ± 0.02 ^Fb^	81.32 ± 0.03 ^Ea^	88.69 ± 0.01 ^Ba^	74.61 ± 0.18 ^Hb^	84.27 ± 0.02 ^Ca^	75.05 ± 0.01 ^Gb^
	FZ	87.71 ± 0.08 ^Ab^	75.88 ± 0.03 ^Eb^	84.86 ± 0.01 ^Bb^	81.98 ± 0.07 ^Ca^	79.27 ± 0.01 ^Db^	86.57 ± 0.03 ^Ab^	84.48 ± 0.05 ^Ba^	84.55 ± 1.76 ^Ba^	79.16 ± 0.00 ^Da^
a*	VC	−1.28 ± 0.01 ^Ia^	1.45 ± 0.01 ^Eb^	−0.780 ± 0.000 ^Ga^	2.22 ± 0.01 ^Ca^	2.48 ± 0.01 ^Ba^	−1.22 ± 0.01 ^Ha^	1.71 ± 0.01 ^Da^	1.13 ± 0.01 ^Fa^	2.69 ± 0.01 ^Aa^
	FZ	−1.67 ± 0.00 ^Hb^	2.85 ± 0.02 ^Aa^	−1.32 ± 0.01 ^Gb^	−0.870 ± 0.010 ^Eb^	0.677 ± 0.032 ^Bb^	−1.75 ± 0.00 ^Ib^	−1.05 ± 0.02 ^Fb^	−0.567 ± 0.015 ^Db^	0.457 ± 0.006 ^Cb^
b*	VC	11.62 ± 0.01 ^Ib^	20.01 ± 0.01 ^Eb^	13.21 ± 0.01 ^Hb^	26.71 ± 0.01 ^Aa^	21.52 ± 0.03 ^Db^	13.69 ± 0.01 ^Ga^	25.27 ± 0.01 ^Ba^	19.43 ± 0.02 ^Fa^	23.90 ± 0.01 ^Ca^
	FZ	12.20 ± 0.05 ^Ha^	28.73 ± 0.06 ^Aa^	14.10 ± 0.01 ^Ga^	18.26 ± 0.06 ^Db^	22.30 ± 0.04 ^Ba^	10.82 ± 0.04 ^Ib^	17.06 ± 0.01 ^Fb^	17.24 ± 0.02 ^Eb^	19.24 ± 0.01 ^Cb^
Solubility (s)	VC	22.05 ± 0.07 ^Fa^	23.47 ± 0.10 ^Ea^	24.41 ± 0.16 ^Ca^	18.09 ± 0.09 ^Ia^	20.26 ± 0.06 ^Ha^	25.05 ± 0.07 ^Ba^	23.97 ± 0.05 ^Da^	27.44 ± 0.20 ^Aa^	21.25 ± 0.07 ^Ga^
	FZ	14.62 ± 0.53 ^Ab^	11.60 ± 0.52 ^Bb^	10.09 ± 0.01 ^Cb^	11.36 ± 0.37 ^Bb^	7.27 ± 0.05 ^Eb^	7.10 ± 0.07 ^Eb^	10.52 ± 0.16 ^BCb^	8.75 ± 0.05 ^Db^	5.28 ± 0.04 ^Fb^
Density (g/mL)	VC	0.844 ± 0.000 ^Aa^	0.785 ± 0.000 ^Da^	0.838 ± 0.000 ^Ba^	0.628 ± 0.000 ^Ha^	0.677 ± 0.000 ^Ga^	0.727 ± 0.000 ^Fa^	0.836 ± 0.001 ^Ca^	0.771 ± 0.000 ^Ea^	0.770 ± 0.000 ^Ea^
	FZ	0.626 ± 0.000 ^Ab^	0.544 ± 0.021 ^Bb^	0.501 ± 0.002 ^BCb^	0.514 ± 0.018 ^BCb^	0.498 ± 0.008 ^CDb^	0.537 ± 0.018 ^BCb^	0.627 ± 0.000 ^Ab^	0.456 ± 0.000 ^Db^	0.455 ± 0.000 ^Db^
Hygroscopicity index (%)	VC	9.88 ± 0.74 ^Ca^	12.80 ± 0.62 ^ABa^	10.33 ± 0.83 ^BCa^	13.81 ± 1.06 ^Aa^	8.24 ± 1.94 ^Ca^	9.54 ± 0.65 ^Ca^	13.51 ± 0.66 ^Aa^	10.66 ± 0.28 ^BCa^	8.83 ± 0.22 ^Ca^
	FZ	3.32 ± 0.39 ^ABb^	2.92 ± 0.03 ^Bb^	3.46 ± 0.17 ^ABb^	3.35 ± 0.23 ^ABb^	3.57 ± 0.29 ^ABb^	3.10 ± 0.19 ^ABb^	3.15 ± 0.15 ^ABb^	3.57 ± 0.29 ^AbB^	3.66 ± 0.22 ^Ab^

LVP: lavender honey powder; THP: thyme honey powder; VP: vetch honey powder; MP: multifloral honey powder; VC: vacuum drying; FZ: freeze drying. ^A–I^: different letters show significant differences (*p* < 0.05) among the different botanical origins. ^a,b^: different letters show significant differences (*p* < 0.05) between drying procedures.

**Table 4:** Glass transition temperature *T*_g_ (midpoint temperature °C) of raw honeys (thymus, lavender, multifloral, vetch) and honey powders obtained with freeze (FZ) and vacuum (VC) drying using maltodextrin (MD) (*n* = 1).

	LV1	LV2	LV3	TH1	TH2	V1	M1	M2	M3
*T*_g_1	−26.00	−28.51	−29.51	−25.81	−29.37	−25.45	−31.31	−37.37	−25.98
*T*_g_2	---	---	---	---	---	---	---	---	---
	FZ-LVP1	FZ-LVP2	FZ-LVP3	FZ-THP1	FZ-THP2	FZ-VP1	FZ-MP1	FZ-MP2	FZ-MP3
*T*_g_1	12.58	16.50	15.28	14.36	15.89	14.26	14.41	15.92	15.97
*T*_g_2	85.75	85.80	85.43	83.03	81.69	92.00	78.34	85.10	83.22
	VC-LVP1	VC-LVP2	FZ-LVP3	FZ-THP1	FZ-THP2	VC-VP1	VC-MP1	VC-MP2	VC-MP3
*T*_g_1	28.09	27.75	34.12	31.39	35.50	31.50	34.27	34.70	34.77
*T*_g_2	85.39	81.87	91.84	---	92.02	94.84	94.51	94.56	87.88

LV: lavender honey; TH: thyme honey; V: vetch honey; M: multifloral honey; LVP: lavender honey powder; THP: thyme honey powder; VP: vetch honey powder; MP: multifloral honey powder; VC: vacuum drying; FZ: freeze drying. ---: absent (there is no *T*_g_2).

#### 3.2.2. Moisture and Water Activity (a_w_)

Table 3 shows the results of moisture and a_w_. Moisture was lower in VC-P (57% honey) than in FZ-P (73%), unlike the data described by Samborska et al. [13], where P with higher honey concentrations exhibited the lowest moisture percentages. Our values were similar to those of Nurhadi et al. [16] for VC-P (1.1%) in samples of 50% honey obtained with VC using MD, and lower than the results obtained in buckwheat honey powders at 50% (MD-FZ) by Keke and Cinkmanis [37] (5.6%). Mutlu and Erbas [1] obtained a mean moisture value of 3.15% in MD-P regardless of the process used (VC or SP) or the honey content (50, 57 or 67%), this percentage being considerably higher than the percentages in our research. Differences with other studies could be mainly due to the honey concentration. Among the FZ-P, no significant differences (*p* ≥ 0.05) were observed considering the different botanical origins. However, regarding VC-P, significant differences were observed considering the origins of honeys, LVP1, LVP2, LVP3, THP1 and MP1 being the powders with the lowest moisture percentages. Therefore, botanical origins, drying procedures and their interaction have an influence on moisture, as shown by multifactor ANOVA (Table 5A). The reduction in moisture during the drying processes when making P significantly contributed to a greater stability of honey during storage [4].

Our VC-P a_w_ values were similar to those obtained by Mutlu and Erbas [1], regardless of the carrier used (0.32), also being similar to a_w_ obtained with MD, regardless of the drying procedure (0.24). Our FZ-P a_w_ values were similar to the value obtained in multifloral honey P obtained using FZ (0.215) by Ganaie et al. [36]. In general, a_w_ was somewhat higher in VC-P, depending on the type of honey, the time elapsed until the analysis and the storage conditions [11]. As for the thermal desorption graphs of P, it was expected that the higher the moisture content, the higher the water activity [38,39], but according to Nurhadi et al. [38], this increase is minimal and not very significant with values of a_w_ below 0.35 (limit value). This fact, together with the differences in composition, might explain that, regarding the same drying process, samples with lower moisture show higher water activities. The discordant results of the same sample subjected to different drying methods could be due to the different proportions of honey:maltodextrin or to different steric effects in the powdered honeys obtained using both methods. Nevertheless, a_w_ did not depend on the drying procedure and honey concentration or the botanical origin of honeys (Table 5A). VC reduced the water activity between 56% and 67% and FZ between 55% and 62%, these percentages being higher than the value obtained by Nedić et al. [4] (28%). Consequently, our P could be less susceptible to microbial growth, avoiding the possibility of fermentation, since most microorganisms cannot multiply below 0.900 a_w_ and cell division of extremophile species is prevented below 0.61 a_w_ [40].

#### 3.2.3. Colour

Table 3 shows the CieLAB colour space tristimulus values of the P obtained using FZ and VC.

For VC and FZ drying methods, the highest L* value was obtained in sample LVP1, which showed the lightest colour intensity in comparison with the samples from other botanical origins. After drying, all L* values of P were higher than L* values of the raw honeys because, during the drying process, samples were lightened, tending towards a whitish colour, probably related to MD addition.

All a* values of P were lower than those of raw honeys (Table 2), except in LVP2. In general, VC-P exhibited higher a* values than FZ-P, tending to show reddish colours, probably due to Maillard reactions, fructose caramelization and polyphenol deterioration reactions at 60 °C [41].

All b* values of P tended towards yellow. As occurred for a*, the b* values of P were also lower than those of the raw honeys (Table 2), mainly because of the addition of MD (dilution factor), except for LVP2 and MP3 made using VC and FZ. LV2 and M3 were the honeys with the lowest b* values (Table 2), so the drying procedure affected the final b* values of their powders.

Values obtained for L*, a*, b* of VC-P were similar to those obtained by Nurhadi et al. [42] in samples obtained with VC using MD (L* = 90.39; a* = 0.06; b* = 14.29). In contrast, our results obtained for FZ-P were slightly different from those of Ganaie et al. [36] (L* = 90.84; a* = 0.01; b* = 12.52).

The botanical origin of honeys and its interaction with the drying procedure– honey concentration were the factors that demonstrated an influence on L*, a* and b* values. The drying method–honey concentration only influenced a* values (Table 5A). The final colour of honey powder depended on the initial colour of the honey, clearly influenced by the botanical origin. The influence of the interaction of the drying procedure–honey concentration and the botanical origin could be explained by the fact that the final colour also depended on the content of Maillard compounds, which was related to the temperature of the procedure, the content of the honey in the original sample (which, in turn, depended on the drying procedure–honey concentration) and the sugar´s composition in the original sample (which, in turn, depended on the botanical origin). The influence of the three factors was observed more intensely in the value of a* (red colour) since Maillard compounds are brownish with an intense influence of red. The colour of the raw honey proved to be the main factor that influenced the parameters L* and b*, being related to the botanical origin and the amount of honey, which explained the influence of the interaction between the botanical origin and drying procedure–honey concentration.

#### 3.2.4. Solubility and Tapped Density

Our solubility times were lower than those described by Nurhadi et al. [16] (MD-VC: 1.08 min) and slightly higher than those (15.74 s) obtained by Nurhadi et al. [42] in another trial using the same conditions. FZ-P showed higher solubility values in comparison with VC-P and the solubility data of other researchers. Therefore, the drying method–honey concentration demonstrated a great influence on the dissolution of samples in water, as well as the botanical origin of samples, and the interaction between both factors (Table 5A). Unlike what other authors such as Samborska and Biénskowska [43] indicated, in this study, the moisture content did not influence the solubility behaviour of samples, because our results showed that even although most assessed honeys had a similar moisture percentage, their solubility was different (r = −0.3089; *p* = 0.0676).

The tapped density was higher in VC-P than in FZ-P, with a positive strong correlation with solubility (r = 0.8824; *p* = 0.000), which was also described by Mutlu and Erbas [1] in P obtained using VC and SP. Similar values were described by Ganaie et al. [36] in P obtained with FZ using MD (0.52 g/mL), and Nurhadi et al. [42] in P obtained with VC using MD (0.80 g/mL). Mutlu and Erbas [1] also obtained similar values for P made with VC (0.72 g/mL). Within the VC-P, LV-P presented the highest tapped density, followed by M-P, V-P and finally TH-P. In the case of honey powders obtained with FZ, there was no clear trend based on botanical origin. In previous studies comparing the same drying procedure, it was observed that the tapped density increased as the concentration of honey increased or the concentration of carrier decreased [44]. However, the results of this study demonstrated that the tapped density was more influenced by the drying procedure than by the honey concentration, since instead of FZ-P (73% honey), VC-P (57% honey) were the honey powders that showed the highest tapped density values. This influence of the drying procedure on tapped density can be explained due to differences in the porosity of the powder particles formed by each procedure. Furthermore, Shi et al. [18] and Mutlu et al. [39] claimed that the higher the moisture content in food, the lower the tapped bulk density. The claim agrees with our results because, in our case, FZ samples were the samples with the highest moisture content, shown by Pearson correlation (r = −0.5534; *p* = 0.0011). Thus, greater spaces for storing, packaging and distributing were necessary for FZ-P, although FZ-P could be less susceptible to phenomena of cohesion and agglomeration [11]. The botanical origin and the drying methods were factors that influenced the density of P and, to a lesser extent, the interaction of these two factors (Table 5A).

#### 3.2.5. Hygroscopicity

P is highly hygroscopic in nature due to the high moisture absorption capacity of fructose [5]. FZ-P showed a lower (*p* < 0.05) hygroscopic index (2.92–3.66%) than VC-P (8.24–13.81%) (Table 4). The drying method–honey concentration proved to influence hygroscopicity (Table 5A). VC-P results were similar to those obtained by Nurhadi et al. [16] (12.0%) and lower than the values obtained by Mutlu and Erbas [1] in honey powders dehydrated by VC (14.56%). In multifloral honey powders dried with FZ using MD, Ganaie et al. [36] described a 28.67% hygroscopic index, which was considerably higher than our values. The hygroscopic rate (amount of water gained per minute) ranged in VC-P from 2 × 10^−5^ to 1.9 × 10^−4^ g H_2_O/min and in FZ-P from 4 × 10^−5^ to 6 × 10^−5^ g H_2_O/min. During the first 4 h, VC-P gained water faster than the FZ-P. Nurhadi et al. [16] obtained a value of 2.4 × 10^−4^ g H_2_O/min for P made with VC using MD.

Shi et al. [18] observed an inverse correlation between moisture and P hygroscopicity. The same correlation was observed in this study, although it was very weak (r = −0.4522, *p* = 0.001). A moderate correlation was noticed between hygroscopicity and density (r = 0.6584, *p* = 0.0001) and between hygroscopicity and solubility time (r = 0.6775, *p* = 0.0001). Thus, samples with higher hygroscopicity levels exhibited higher compactibility and lower solubility.

### 3.3. Potentially Bioactive Properties of Honey Powders

#### 3.3.1. Total Phenolic Content (TPC) and Total Flavonoid Content (TFC)

**Figure 1:**
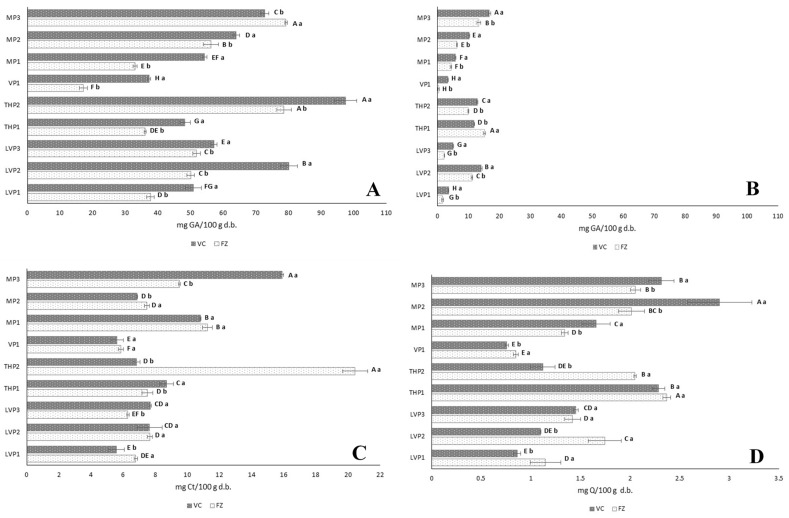
Total phenols (mg of GA/100 g dry basis (d.b.)) of honey powders (**A**) and their methanolic extracts (**B**), total flavanols (mg of Ct/100 g dry basis (d.b.)) (**C**) and total flavone/flavonols (mg of Q/100 g dry basis (d.b)) (**D**) obtained with vacuum (VC) and freeze (FZ) drying using maltodextrin (MD) (*n* = 3). Error bars represent the standard deviation for each data point. Different capital letters (A–H) indicate significant differences (*p* < 0.05) among different honeys. Different lowercase letters (a,b) indicate significant differences (*p* < 0.05) between drying procedure. Ct: catechin; Q: quercetin; LVP: lavender honey powder; THP: thyme honey powder; VP: vetch honey powder; MP: multifloral honey powder.

Figure 1A shows the TPC of P. VP1 showed the lowest TPC values for both VC-P (37.37 mg GA/100 g d.b.) and FZ-P (17.19 mg GA/100 g d.b.). THP2 exhibited the highest values for both VC (97.54 mg GA/100 g d.b) and FZ (78.67 mg GA/100 g d.b.), although FZ-THP2 did not show significant differences in comparison with FZ-MP3. On the other hand, a higher TPC was observed in VC-P than in FZ-P (except for MP3), although the honey content was higher in FZ-P (73% honey) compared to VC-P (57% honey). This higher TPC was possibly due to the Maillard compounds formed during the heating process (60 °C/48 h), which could have an influence on the colour measured at 734 nm. Although no clear trend based on the botanical origin of the honeys was observed, both the drying procedure–honey concentration and the botanical origin of the honey significantly influenced the TPC (Table 5B). Keke and Cinkmanis [37] obtained higher TPC in powdered buckwheat honeys made with MD using FZ (92–130 mg GA/100 g d.b.), and Osés et al. [21] also obtained higher values in powdered heather honeys (LHP) with MD made using VC (29.64 to 143.95 mg GA/100 g d.b.) and FZ (74.35 to 149.72 mg GA/100 g d.b.). The TPC of P was lower than the TPC of the raw honeys, with the exception of VC-VP1 (Table 2). P demonstrated to be able to retain between 49.7 and 114.2% of the phenolic compounds, keeping the polyphenols present in the honeys. Methanolic extracts’ TPC (TPC-E) was assayed in order to remove the interference of other reducing substances (i.e., sugars) that react with Folin–Cioucalteu reagent in honey TPCs. As was expected, TPC-E was lower than TPC (Figure 1B). The VC-P methanolic extracts showed the highest TPC (3.45–16.86 mg GA/100 g d.b.) in respect of the FZ-P extracts (0.411–15.30 mg GA/100 g d.b.), with the exception of sample THP1. As expected, the VP1 sample showed the lowest TPC-E. MP3 was the powder with the highest TPC-E regarding VC, whereas THP1 was the powder with the highest TPC-E regarding FZ. TPC-E was influenced by the botanical origin of honeys (Table 5B).

Flavanols (TFCC) ranged (Figure 1C) from 5.39 to 20.45 mg Ct/100 g d.b., while flavone/flavonols (TFCQ) (Figure 1D) ranged from 0.757 to 2.90 mg Q/100 g d.b., the values being higher than those obtained for the raw honeys (Table 2). VC-VP1 and FZ-VP1 exhibited the lowest TFC (both for TFCC and TFCQ). In general, there were no significant differences in TFCC considering the drying method–honey concentration. However, concerning TFCQ, VC-MP showed higher contents, while for the rest of the honey powders, FZ samples exhibited higher contents. Multifactor ANOVA showed that, unlike the drying method, the botanical origin influenced TFC (Table 5B). The ranges of both TFCC and TFCQ were lower than those obtained by Osés et al. [21] in LHP (0.38–36.3 mg Ct/100 g b.d. and 0–3.6 mg Q/100 g b.d.). In comparison with the raw honeys (Table 2), the TFCC values of the honey powders were higher, probably due to the interference of MD.

#### 3.3.2. Antiradical Activities

**Figure 2:**
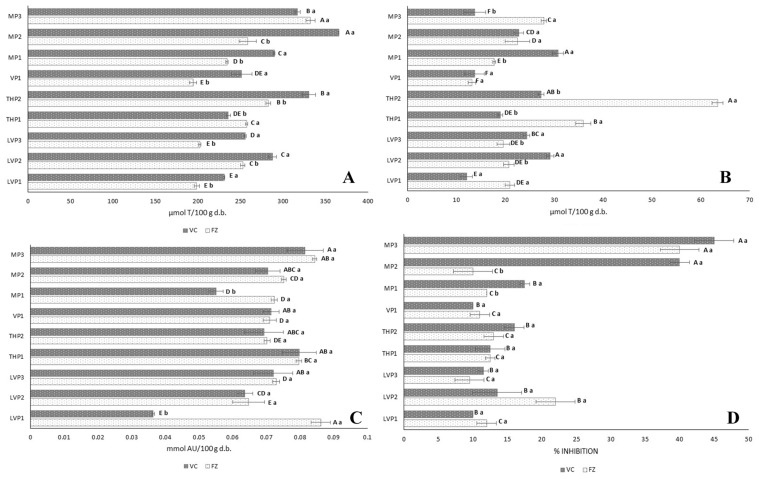
Antioxidant activity of honey powders obtained with freeze (FZ) and vacuum (VC) drying, using maltodextrin (MD) against different radicals (ABTS^•+^ (**A**), ROO^•^ (**B**), ^•^OH (**C**) and O_2_^•−^ (**D**)) (*n* = 3). Error bars represent the standard deviation for each data point. Different capital letters (A–F) indicate significant differences (*p* < 0.05) between the different botanical origins. Different lowercase letters (a,b) indicate significant differences (*p* < 0.05) between drying procedures. LVP: lavender honey powder; THP: thyme honey powder; VP: vetch honey powder; MP: multifloral honey powder.

Figure 2 shows the antiradical activities of P. TEAC (Trolox equivalent antioxidant capacity) (Figure 2A), against ABTS^•+^, ranged between 230.83 and 365.58 μmol T/100 g d.b. for VC-P, and between 194.18 and 332.25 μmol T/100 g d.b. for FZ-P. The lowest TEAC values were observed in VC-LVP1 and FZ-VP1. No significant differences (*p* < 0.05) were found for samples VC-THP1- and VC-VP1 and for samples FZ-LVP1 and FZ-LVP3. VC-MP2 and FZ-MP3 exhibited the highest TEAC. Generally, VC-P obtained higher TEAC than FZ-P, probably due to Maillard compounds, which could contribute to an increase antioxidant activities [45]. The TEAC values of P were higher than TEAC values of raw honeys (Table 2). MD exhibited a high TEAC value (193.43 μmol T/100 g d.b.) [11], contributing to the anti-ABTS^•+^ radical of P. Our TEAC values were similar to those reported by Mutlu and Erbas [1] for blossom honey powders obtained with MD by both VC and SP techniques (251 μmol T/100 g), and by Samborska et al. [19] for powders obtained using spray drying, also using MD (726 mg T/Kg = 290 μmol T/100 g solids). Our TEAC values were lower than those of VC-LHP and FZ-LHP obtained by Osés et al. [21] (317–673 μmol T/100 g d.b), using MD. TEAC was shown to be influenced by the drying method–honey concentration and the botanical origin of the honeys (Table 5B), the latter influence also being described by Rivero et al. [2]. The oxygen radical absorbance capacity (ORAC) against ROO^•^ (Figure 2B) ranged from 12.05 to 30.77 μmol T/100 g d.b. for VC-P, while for FZ-P, it ranged from 13.15 to 63.47 μmol T/100 g d.b. An ORAC value of 15.21 μmol T/100 g was obtained for MD [11]. ORAC was higher for most of the honeys in comparison with P (Table 2). Our ORACs were similar to the capacities obtained by Osés et al. [21] in VC-LHP and FZ-LHP using MD (13.40–55.88 μmol T/100 g d.b). ORAC was demonstrated to be highly influenced by the botanical origin of the honeys, the drying method–honey concentration, and the interaction of both factors (Table 5B).

The hydroxyl radical-scavenging activity (AOA) (Figure 2C) ranged between 0.036 and 0.082 mmol AU/100 g d.b. for VC-P and between 0.065 and 0.086 mmol AU/100 g d.b. for FZ-P. MD showed an AOA of 0.160 mmol AU/100 g [21], thus influencing the activity of the samples. Neither the botanical origin nor the drying procedure significantly influenced the AOA (Table 5B). In this work, the AOA results were higher than those of the LHP obtained with VC and FZ using MD (0.037–0.061 mmol AU/100 g d.b.) [21].

The superoxide radical-scavenging activity (SRS) (Figure 2D) ranged between 7 and 45% for VC samples and between 9.5 and 40% for FZ samples, the inhibition percentage of MD being 5.5%. For both VC and FZ, the highest inhibition was shown by MP3. No differences were found between the SRS of P obtained with VC and FZ (*p* ≥ 0.05), except for the samples MP1 and MP2, whose SRS results were higher in VC samples. The SRSs were lower in P than in the raw honeys (Table 2). In general, the SRSs were also lower than the anti-O_2_^•−^ activity of LHP obtained with VC and FZ using MD (11–88%) [21]. SRS was influenced by the botanical origin of honeys, but not by the drying method (Table 5B).

These results show that the antiradical activity of P against different radicals is different and depends on different compounds in the honey, which may vary quantitatively or qualitatively from one honey to another and which may or may not be modified by the drying process. Statistical analysis shows a positive correlation between the activities against ABTS^•+,^ ROO^•^ and O_2_^•−^ radicals with the total phenols and flavonoids (r between 0.329 and 0.6298; *p* < 0.05), while there is no correlation between these compounds and the activity against the -OH radical (*p* > 0.05).

#### 3.3.3. Anti-Inflammatory Activity

**Figure 3:**
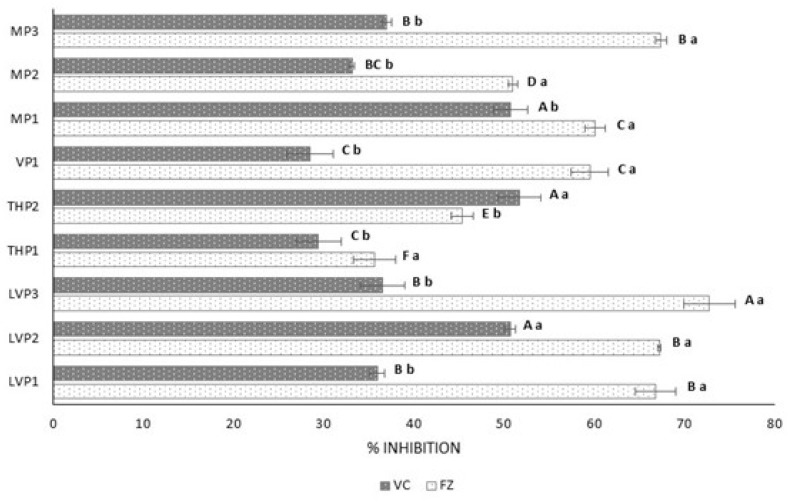
Anti-inflammatory activity of powdered honeys obtained with vacuum (VC) and freeze (FZ) drying, using maltodextrin (MD) (*n* = 3). Error bars represent the standard deviation for each data point. Different capital letters (A–F) indicate significant differences (*p* < 0.05) among the different botanical origins. Different lowercase letters (a,b) indicate significant differences (*p* < 0.05) between drying procedures. LVP: lavender honey powder; THP: thyme honey powder; VP: vetch honey powder; MP: multifloral honey powder.

The anti-inflammatory activity of P (Figure 3) varied between 28.53 and 51.72% (VC) and between 35.69 and 72.81% (FZ). MD showed no hyaluronidase inhibition [11]. The % inhibition rates were higher in FZ samples (73% honey) than in VC samples (57% honey), except for THP2. In comparison with the raw honeys (Table 2), the anti-inflammatory activity was similar for FZ-P and lower for VC-P. The anti-inflammatory activity was affected by the drying method–honey concentration, the botanical origin of honeys and the interaction of both factors (Table 5B), appearing to be strongly affected by the honey concentration. However, this activity was not correlated with total phenols or total flavonoids (*p* > 0.05).

#### 3.3.4. Antimicrobial Activities

**Figure 4:**
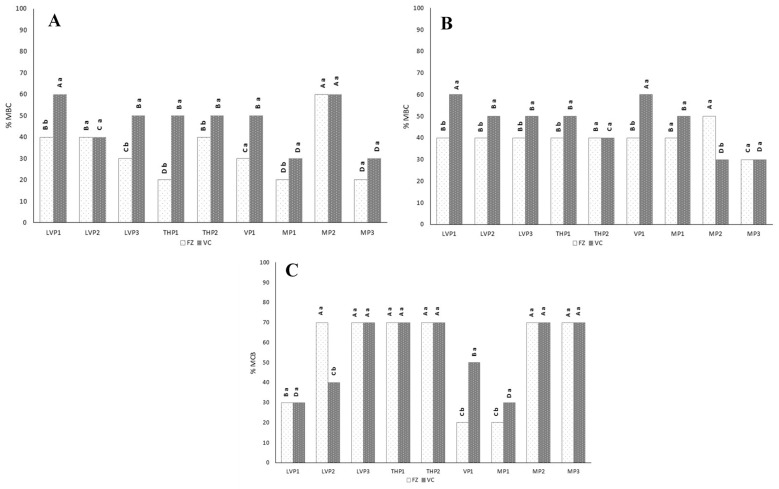
Minimal bactericidal concentration (MBC) of honey powders obtained with vacuum (VC) and freeze (FZ) drying, using maltodextrin (MD) expressed as % honey against *St. aureus* (**A**), *E. coli* (**B**) and *L. monocytogenes* (**C**) (*n* = 3). Triplicates show identical MBCs for each sample. Different capital letters (A–D) indicate significant differences (*p* < 0.05) among the different botanical origins. Different lowercase letters (a,b) indicate significant differences (*p* < 0.05) between drying procedure. LVP: lavender honey powder; THP: thyme honey powder; VP: vetch honey powder; MP: multifloral honey powder.

Figure 4 shows the results of the antimicrobial activity of P at a concentration range between 5 and 70%, after using the broth microdilution method. MD showed no antimicrobial activity for the tested concentrations [21]. P showed an MBC between 20 and >70%. Overall, *L. monocytogenes* (Figure 4C) was the most resistant microorganism, while *St. aureus* (Figure 4A) was the most sensitive for most samples. However, for LVP1, VP1 and MP1, *L. monocytogenes* was the most sensitive microorganism. The antimicrobial activities against *St. aureus* and *E. coli* (Figure 4A,B) were similar or lower in VC-P (57% honey) than in FZ-P (73% honey), except for MP2, regarding *E. coli*, in which MBC was higher for FZ-MP2 than for VC-MP2. This fact could be due to the heating process involved during VC, with possible losses of some volatile compounds with antimicrobial activity and also due to the lower concentration of honey in VC-P. Tomczyk et al. [46] led to the same conclusion, not having obtained antibacterial activity in honey powders made with SP using MD, in this case due to losses of honey thermolabile compounds, and to the 50% powder dilution with MD. However, P showed different behaviour for *L. monocytogenes*, the MBC values of VC-P and FZ-P being similar, except for LVP2, VP1 and MP1, whose MBCs were also lower for VC-P.

For most samples, the MBCs of P were higher than those of the raw honeys (Table 2), thus exhibiting lower antimicrobial activity. The MBCs for *St. aureus* and *E. coli* were influenced by the drying method–honey concentration. The MBCs for *L. monocytogenes* and *E. coli* were influenced by the botanical origin of samples.

### 3.4. Statistical Analysis

**Figure 5:**
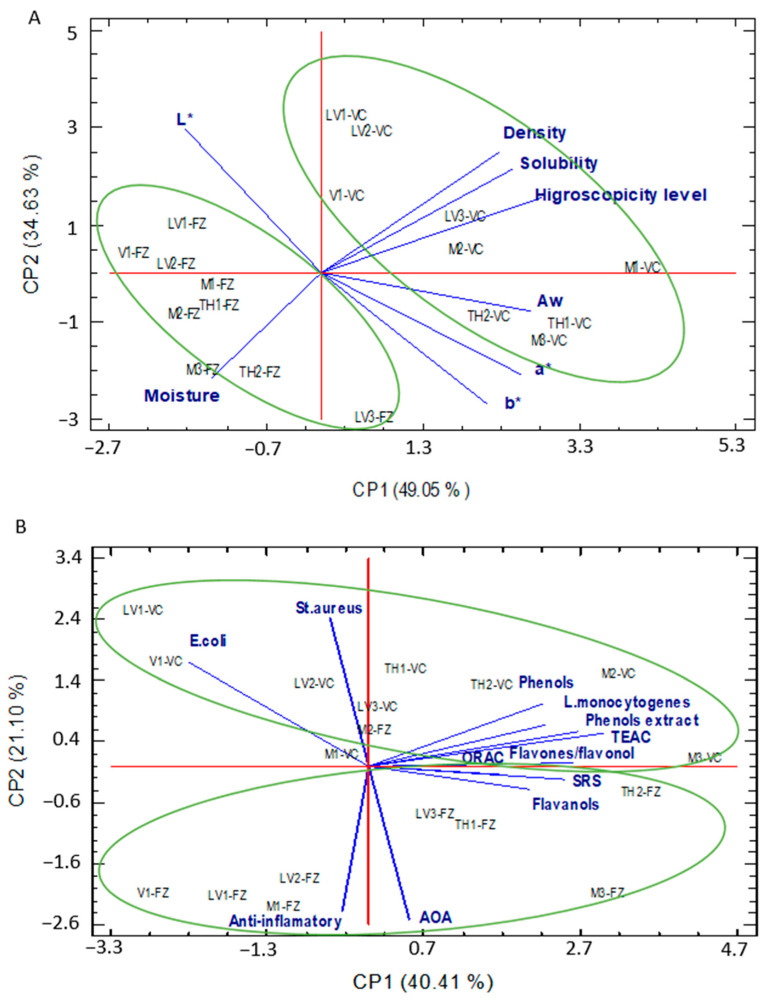
Principal component analysis of physicochemical properties (**A**) and potentially bioactive properties (**B**) of honey powder obtained with vacuum drying (VC) and freeze drying (FZ). LVP: lavender honey powder; THP: thyme honey powder; VP: vetch honey powder; MP: multifloral honey powder.

Two PCAs were carried out: one on the physico-chemical results and the other one on the bioactive properties. Regarding the PCA on the physico-chemical properties (Figure 5A), the first component explained 49.05% of the variance, while the second component explained 34.63%. PC1 was mainly defined by hygroscopicity and a_w_, while PC2 was mainly defined by L*. P were separated into two groups. The first one was composed by VC-P (on the right side of Figure 5A), and the second one (on the left side of Figure 5A) by FZ-P. VC-P was characterized by higher density, hygroscopicity a_w_ and a*, as well as lower solubility and moisture than FZ-P. With regard to the PCA about bioactive properties (Figure 5B), the first component explained 40.41% of the variance and the second component 21.10%. PC1 was defined by TPC, TFC, SRS, TEAC and ORAC, in contrast to PC2, which was defined by anti-inflammatory activity, AOA and antimicrobial activity against *St. aureus*. Again, the powders were separated into two groups. The first one (in Figure 5B, above) was composed of all VC-P and the second one (in Figure 5B, below) was composed of most FZ-P, the latter being characterized by higher AOA and anti-inflammatory activities, as well as by higher antimicrobial activity against *St. aureus* and *E. coli*. Although PCA separated the samples by the drying procedure–honey concentration factors, the botanical origin also showed an influence (Table 5). This influence was also described by Tomczyk et al. [46], who stated that the quality of honey powder was directly related to the botanical origin of the honey used for honey powder production.

## 4. Conclusions

Most P showed lower TPC, ORAC, SRS and antimicrobial activities than their corresponding raw honey, whereas TFC and TEAC were higher in P. FZ provided the highest recovery, with a higher final honey concentration in P than VC. FZ-P showed higher moisture and solubility and lower density and hygroscopicity than VC-P. The *T*_g_, moisture, hygroscopicity, solubility, density, TPC, TEAC, ORAC, anti-inflammatory and antimicrobial activities against *St. aureus* and *E. coli* depended on the drying method (using the most appropriate honey–maltodextrin concentration for each drying technique). Moisture, colour, solubility, density, TPC, TEAC, ORAC, SRS, and anti-inflammatory and antimicrobial activities against *L. monocytogenes* and *E. coli* depended on the botanical origin of the honey. Our results show that honey powder is a promising substitute for honey in food as well as in the pharmaceutic and cosmetic industries because it keeps the advantages of the raw honey, being easier to transport, store and use. In addition, consumers and markets are increasingly hard to please, demanding natural products with the minimum possible quantity of additives. Honey dried using vacuum drying and freeze drying with a natural ingredient (maltodextrin) can be kept at low temperatures without the need to further add anti-caking agents, so they could be labelled with a “Clean Label” as “Real Food”, helping to improve their commercialization.

## Figures and Tables

**Table 1:** Botanical origins of honey samples.

Honey	Family	Scientific Name	Common Name
TH1	Labiatae	*Thymus* sp.	Thyme
TH2	Labiatae	*Thymus* sp.	Thyme
LV1	Labiatae	*Lavandula hybrida* Reverchon	Lavender
LV2	Labiatae	*Lavandula latifolia* Medik	Lavender
LV3	Labiatae	*Lavandula stoechas* Lam	Lavender
V1	Leguminosae	*Vicia* sp.	Vetch
M1	Multifloral	-	Multifloral
M2	Multifloral	-	Multifloral
M3	Multifloral	-	Multifloral

**Table 5:** Effect of interaction (multifactor ANOVA) between drying procedures (using the most appropriate honey–maltodextrin concentration for each drying technique) and botanical origin for physicochemical (A) and bioactive (B) properties of powdered honeys.

(A)	Source	Moisture	Hygroscopicity Level	a_w_		L*	a*		b*	Solubility		Tapped Density	*T*_g_1
	A: Drying-honey concentration	**	***	NS		NS	***		NS	***		***	***
	B: Bot. orig.	**	NS	NS		***	***		***	**		***	NS
	Interaction: AB	**	NS	NS		**	**		*	**		*	NS
(B)	Source	TPC	TPC-E	TFC_C_	TFC_Q_	ABTS^•+^	ROO^•^	^•^OH	O_2_^•−^	Anti-infl.	*St. aureus*	*L.* *mono*	*E. coli*
	A: Drying -honey concentration	**	NS	NS	NS	**	**	NS	NS	***	**	NS	***
	B: Bot. Orig.	***	***	***	***	***	***	NS	**	***	NS	**	***
	Interaction: AB	NS	NS	**	**	NS	***	NS	NS	***	NS	NS	***

Bot. orig.: botanical origin; TPC: total phenolic content; TPC-E: total phenolic content in methanolic extracts; TFCC: flavanols; TFCQ: flavone/flavonol; Anti-infl.: anti-inflammatory; *L. mono*: *L. monocytogenes*. * *p*-value < 0.05; ** *p*-value < 0.01; *** *p*-value < 0.001; NS: non-significant.

## Data Availability

The data presented in this study are available on request from the corresponding authors.

## Supplementary Materials

The following supporting information can be downloaded at: https://www.mdpi.com/article/10.3390/foods12213990/s1, Figure S1: Physical properties of honey powders [16,43,47,48,49]; Figure S2: Biological properties of honey powders [21,50,51,52,53,54,55,56]; Table S1: Abbreviation list.
