# Supplementary material for: Impact of the Drying Procedure and Botanical Origin on the Physico-Chemical and Potentially Bioactive Properties of Honey Powders
Source: Foods. 2023 Oct 31;12(21):3990. doi: 10.3390/foods12213990 (PMC10650056; doi:10.3390/foods12213990)
Supplement: Supplementary file 1 [file foods-12-03990-s001.zip › foods-2661313-supplementary.pdf]

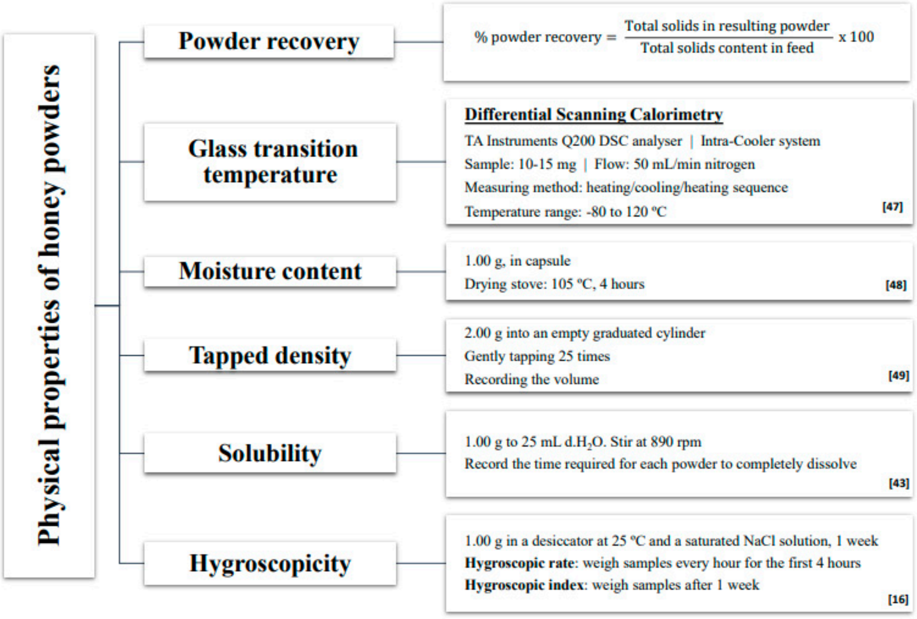

Figure S1. Physical properties of honey powders [16,43,47–49].

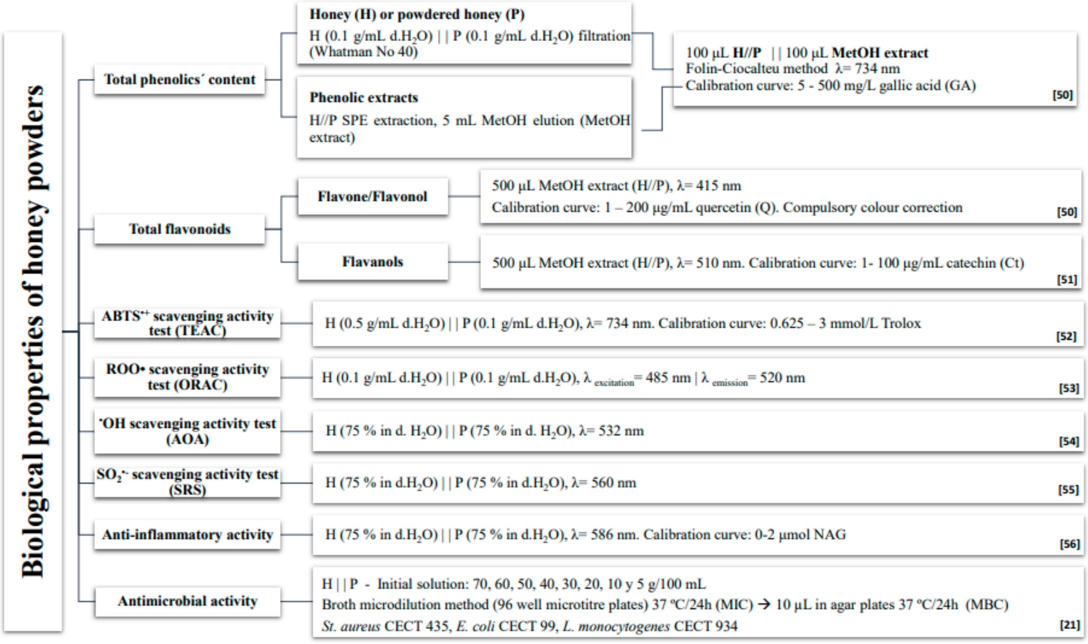

Figure S2. Biological properties of honey powders [21,50–56].

Table S1. Abbreviation's list

ABTS: 2,2´-azino-bis(3-ethylbenzothiazoline-6-sulphonic acid)

ANOVA: Analysis of variance

AOA: Hydroxyl radical-scavenging activity

A_w: water activity

Ct: catechin

FZ: Freeze drying

LHP: Ling-heather honey powders

LV: Lavender honey

LVP: Lavender powder honey

M: Multifloral honey

MBC: Minimal bactericide concentration

MD: Maltodextrin

MP: Multifloral powder honey

ORAC: Oxygen radical absorbance capacity

P: honey powder

PCA: Principal component analysis

Q: Quercetin

SP: Spray drying

SRS: Superoxide radical-scavenging activity

T: Trolox (6-hydroxy-2,5,7,8-tetramethylchroman-2-carboxylic acid)

TEAC: Trolox equivalent antioxidant capacity

TFC: Total flavonoids content

TFC_C: Total flavanols

TFC_Q: Total flavones/flavonols

T_g: Glass transition temperatures

TH: Thyme honey

THP: Thyme powder honey

TPC: Total phenolic content

TPC-E: Total phenolic content in extract

V: Vetch honey

VC: Vacuum drying

VP: Vetch powder honey
